# Multimodal deep learning outperforms clinical and brain region models in predicting stroke-associated pneumonia: an explainable AI study

**DOI:** 10.3389/fneur.2026.1917270

**Published:** 2026-09-14

**Authors:** Haoyang He, Xu Zhang, Lijuan Gu, Zhihong Jian, Xiaoxing Xiong

**Affiliations:** 1 Department of Neurosurgery, Renmin Hospital of Wuhan University, Wuhan, China; 2 Central Laboratory, Renmin Hospital of Wuhan University, Wuhan, China

**Keywords:** Automated Anatomical Labeling Atlas 3, deep learning, image registration, pneumonia, stroke

## Abstract

**Background:**

Stroke-associated pneumonia (SAP) is a frequent complication after acute ischemic stroke (AIS) and is associated with poor outcomes. This study aimed to develop an interpretable multimodal deep learning model integrating MRI, lesion-related brain regions, and clinical variables for early SAP prediction.

**Methods:**

A total of 426 AIS patients were retrospectively enrolled, including 71 patients with SAP. Multimodal MRI data (DWI, T1WI, and T2-FLAIR) were processed using standardized registration and lesion segmentation. A 3D convolutional neural network was used to extract imaging representations, which were fused with clinical variables and AAL3-based brain-region features. Model performance was assessed using stratified five-fold cross-validation and nested cross-validation when applicable, with further evaluation based on receiver operating characteristic (ROC) analysis, calibration analysis, and decision curve analysis. Grad-CAM was applied for model interpretation.

**Results:**

The multimodal fusion model achieved the best performance for SAP prediction, with an AUC of 0.782 (95% CI: 0.712–0.839), compared with the clinical model based on conventional clinical variables (AUC = 0.756), the imaging model based on 3D CNN representations (AUC = 0.693), and the brain-region model based on AAL3-derived lesion location features (AUC = 0.501). The fusion model showed superior clinical utility and favorable calibration. Grad-CAM visualization demonstrated that model predictions were mainly driven by lesion-related cortical and subcortical regions.

**Conclusion:**

A multimodal deep learning framework integrating MRI, brain-region information, and clinical characteristics improved SAP prediction after AIS and provided an interpretable approach for individualized risk stratification.

## 1 Background

As the most common subtype of stroke, acute ischemic stroke (AIS) remains a leading global cause of death and persistent disability (1, 2). The condition arises from a sudden cessation of cerebral perfusion, which subsequently leads to neuronal injury and functional deficits (3). Although substantial progress has been made in acute stroke treatment, complications following stroke continue to have a significant impact on patient prognosis (4). Among these, stroke-associated pneumonia (SAP) is one of the most frequent and clinically relevant complications, with an incidence ranging from 10 to 30% (4, 5). SAP is strongly associated with increased mortality, prolonged hospitalization, and poor functional recovery (6).

The development of SAP is multifactorial and involves complex interactions between neurological injury, immune dysregulation, and impaired protective reflexes such as swallowing and coughing (7). Previous studies have identified several clinical risk factors, including advanced age, stroke severity, dysphagia, and systemic inflammatory markers (8). However, these factors alone are often insufficient for accurate early prediction. Several clinical scoring systems, such as the A^2^DS^2^ and ISAN scores, have been developed to predict SAP risk; however, their predictive performance remains moderate because they rely primarily on clinical variables and do not incorporate imaging or neuroanatomical information. Therefore, incorporating imaging-derived information with clinical variables may provide additional predictive value beyond these conventional scoring systems (9, 10).

Magnetic resonance imaging (MRI) plays a crucial role in the diagnosis and evaluation of AIS, providing detailed information about lesion location, extent, and tissue characteristics, and lesion location has been shown to influence post-stroke complications including infection (3, 11). In recent years, radiomics has gained increasing attention as a promising method for deriving high-dimensional quantitative features from medical images (12, 13). By converting imaging data into structured features, radiomics enables the development of predictive models for various clinical outcomes (13). Several studies have applied radiomics to predict SAP and achieved encouraging performance. However, these approaches primarily rely on handcrafted image features extracted from predefined regions of interest, which may fail to capture complex spatial patterns and multimodal interactions. Furthermore, most existing radiomics studies focus on imaging features alone or combine imaging with limited clinical variables, without explicitly incorporating lesion-aware neuroanatomical information or end-to-end deep feature learning (14).

With the rapid advancement of artificial intelligence, deep learning has shown superior performance in medical image analysis by enabling end-to-end feature learning directly from raw imaging data (14). Convolutional neural networks (CNNs), in particular, can automatically learn hierarchical representations that reflect complex anatomical and pathological characteristics. Compared with traditional radiomics approaches, deep learning reduces reliance on manual feature engineering and enables automatic extraction of hierarchical imaging representations from complex medical images (14).

In addition to imaging features, lesion location and brain functional anatomy play critical roles in the development of SAP. Brain regions such as the insular cortex, precentral gyrus, and cingulate cortex are closely associated with swallowing function, respiratory control, and autonomic regulation (15). Damage to these regions may contribute to dysphagia, impaired airway protection, and the induction of stroke-induced immunodepression syndrome (SIDS), thereby increasing the risk of pneumonia (16, 17). These findings align with the emerging concept of the brain–lung axis, and more recently, the brain–gut–lung axis, which posits that central nervous system injury can disrupt systemic immunity and even gut microbiota, ultimately predisposing patients to pulmonary infection (18, 19). Therefore, integrating lesion-aware anatomical information into predictive models may provide additional clinical value.

In addition, the combination of imaging data with clinical variables has been shown to enhance the predictive performance of models (20–22). Inflammatory markers and coagulation-related parameters serve as indicators of systemic physiological conditions and offer additional information that complements imaging-based features (23). A multimodal fusion strategy that combines MRI data, brain region information, and clinical features may therefore provide a more comprehensive representation of SAP risk. Compared with conventional radiomics pipelines, the proposed multimodal framework enables complementary learning from MRI appearance, lesion location, and clinical characteristics, allowing the model to capture both anatomical and systemic factors associated with SAP development.

Despite these advances, three important limitations remain. First, most existing SAP prediction studies are based on handcrafted radiomics or clinical variables rather than end-to-end deep learning. Second, lesion-aware neuroanatomical information has rarely been integrated into predictive frameworks despite its known association with swallowing dysfunction and autonomic regulation (24–27). Third, few studies have simultaneously combined multimodal MRI, anatomical brain region information, and clinical variables within a unified and interpretable deep learning framework (28, 29).

Therefore, in this study, we propose a multimodal deep learning framework that integrates MRI data, lesion-aware brain region encoding, and clinical variables to predict SAP in patients with AIS. Moreover, model interpretability is improved by applying Gradient-weighted Class Activation Mapping (Grad-CAM) to visualize regions that contribute to the predictions (30, 31). This approach may complement existing clinical SAP scoring systems by providing individualized, imaging-informed risk stratification.

## 2 Materials and methods

### 2.1 Patient population

This retrospective study was conducted in accordance with the Declaration of Helsinki and approved, with the requirement for informed consent waived due to the study design. A total of 516 patients with ischemic stroke treated between May 2022 and October 2023 were initially screened. Eligible participants were adults (>18 years) diagnosed with acute ischemic stroke (AIS) who had complete clinical data and had undergone MRI examinations after admission as well as routine blood tests, with no prior history of radiotherapy or chemotherapy. All clinical variables and laboratory measurements used for model development were collected within the first 24 h after hospital admission and before the clinical diagnosis of stroke-associated pneumonia. The diagnosis of stroke-associated pneumonia (SAP) was established in accordance with the Centers for Disease Control and Prevention (CDC) guidelines (32). SAP diagnosis required fulfillment of clinical symptoms, laboratory evidence of infection, and radiographic findings, and all diagnoses were confirmed through review of the electronic medical records by experienced stroke physicians. Patients were subsequently monitored from admission through completion of treatment, and those who met the predefined diagnostic criteria for Stroke-associated pneumonia during this period were classified as having SAP. Patients were excluded if the imaging data were severely affected by motion artifacts or excessive noise, if Diffusion-weighted imaging (DWI) images lacked clear hyperintense lesions or had inconclusive findings, or if other conditions associated with coagulation disorders were present. Ultimately, 426 patients met the criteria and were enrolled (Figure 1).

**Figure 1:**
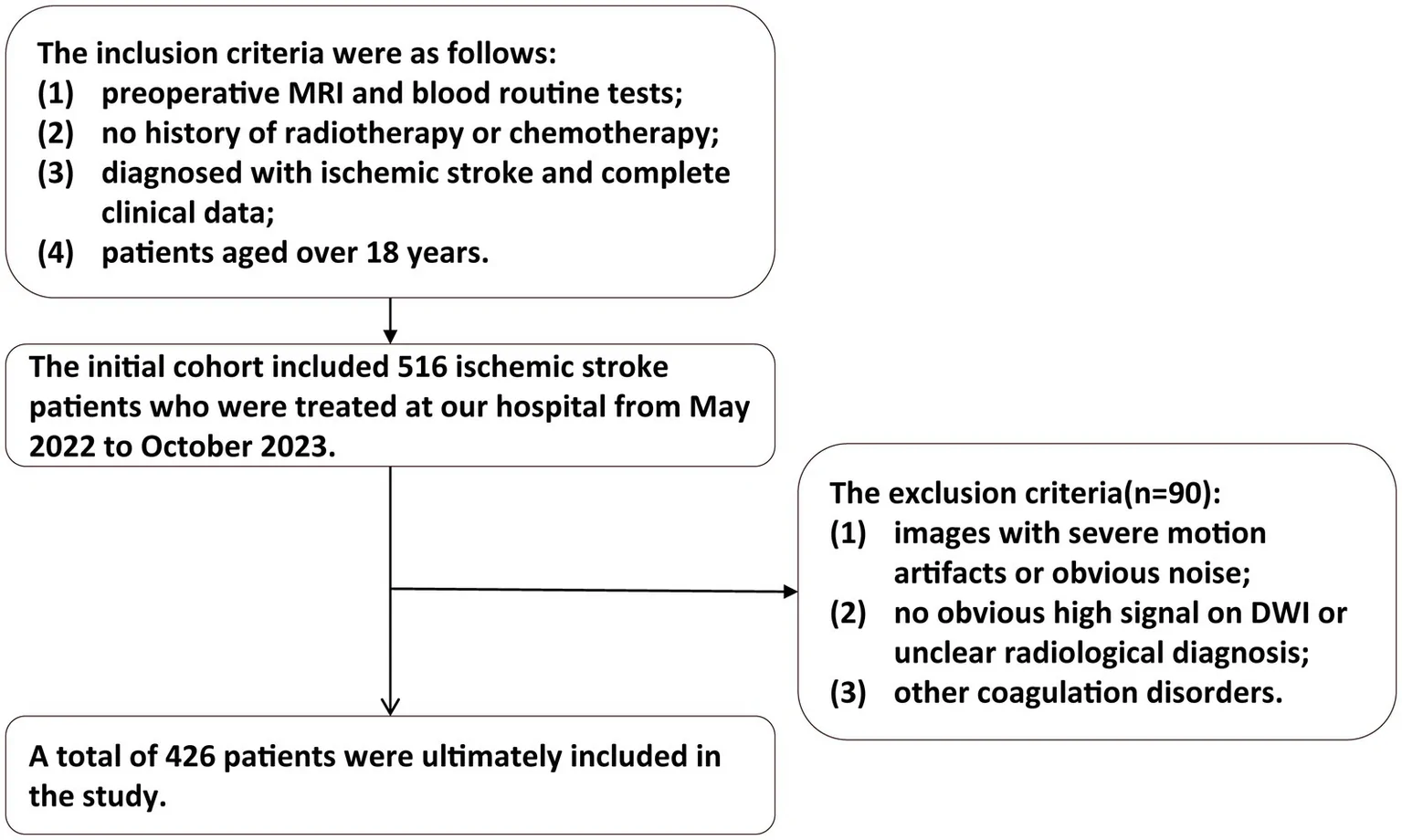
The flow chart of patients’ enrollment. This figure illustrates the complete patient selection process, including initial assessment, exclusion criteria, and the final number of participants included.

### 2.2 MRI protocol

MRI examinations were performed using a GE Discovery 750 3.0 T system (GE Healthcare, Fairfield, United States) within 24 h after stroke onset. To reduce motion-related artifacts, patients were instructed to remain still with their eyes closed. The imaging protocol consisted of DWI, T1-weighted imaging (T1WI), and T2-FLAIR sequences. For axial T1WI, the parameters were TR = 1750 ms, TE = 24 ms, TI = 780 ms, FOV = 240 × 240 mm, and slice thickness = 5.0 mm. Axial T2-FLAIR was acquired with TR = 8,400 ms, TE = 145 ms, TI = 2,100 ms, FOV = 240 × 240 mm, and slice thickness = 5.0 mm. Axial DWI parameters included TR = 3,000 ms, TE = 80 ms, FOV = 240 × 240 mm, slice thickness = 5.0 mm, and *b*-values of 0 and 1,000 s/mm^2^. The acquisition time was approximately 6 min for DWI and 4 min each for T1WI and T2-FLAIR.

Image quality was carefully evaluated, and datasets with head motion exceeding 3.0 mm or rotational displacement greater than 3° were excluded. After removing low-quality images, a total of 426 patients were included in the final MRI analysis cohort, comprising 71 cases with stroke-associated pneumonia (SAP) and 355 without SAP.

### 2.3 Lesion segmentation

Automated lesion segmentation was conducted using the nnU-Net framework (nnUNetv2), a self-configuring deep learning segmentation framework for biomedical image segmentation. DWI scans were used as input to capture acute ischemic lesions.

All images were preprocessed using the nnU-Net automated pipeline, including resampling to a uniform voxel spacing, intensity normalization, and data augmentation strategy optimization. A 3D full-resolution U-Net architecture (3d_fullres) was employed to preserve spatial context and capture volumetric lesion characteristics.

Model training was performed on a GPU using the default nnU-Net configuration, which includes dynamic network adaptation, deep supervision, and patch-based sampling.

The trained segmentation model generated voxel-wise lesion masks for each patient. These masks were subsequently used to extract lesion-based features and to guide downstream multimodal analysis.

### 2.4 Image preprocessing and registration

All MRI modalities, including DWI, T1WI, and T2-FLAIR, were spatially normalized to a common anatomical space using the Automated Anatomical Labeling 3 (AAL3) atlas as the reference template. Image preprocessing and registration were performed using the BRAINSFit module in 3D Slicer (Figure 2).

**Figure 2:**
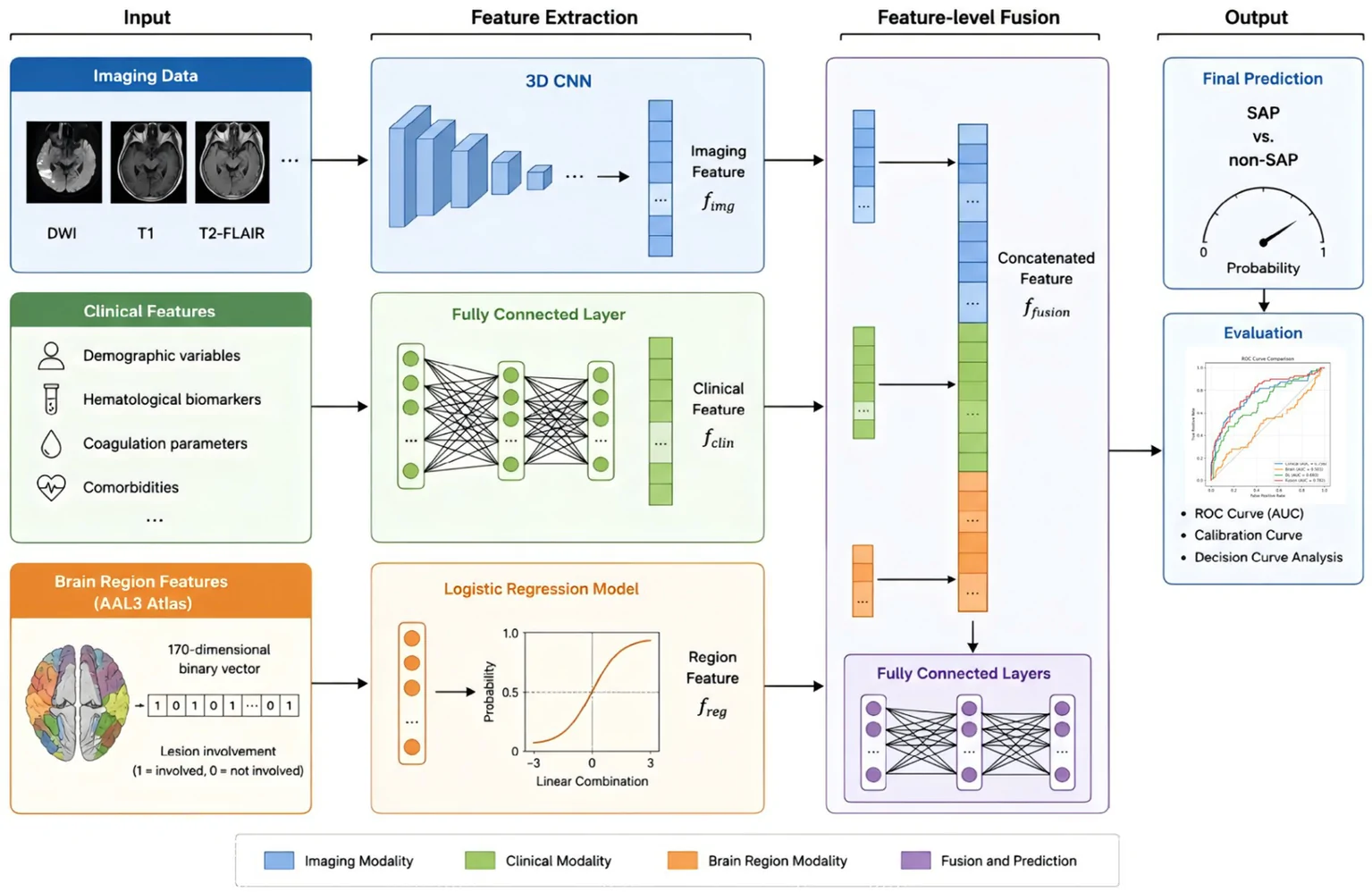
Schematic illustration of the proposed multimodal framework for SAP prediction. MRI-derived imaging features, clinical variables, and lesion-aware brain region features (AAL3 atlas, 170 regions) are extracted and integrated via feature-level fusion. Imaging features are learned using a 3D CNN, clinical features are modeled with fully connected layers, and brain region involvement is encoded as a binary vector and modeled using logistic regression. The fused features are then used to generate the final prediction.

For each subject, DWI was selected as the anchor modality due to its sensitivity to acute ischemic lesions. Linear registration was performed using a multi-stage strategy, including rigid, similarity (scale), and affine transformations. Initialization was conducted using moments-based alignment, followed by iterative optimization with mutual information-based metrics.

The estimated transformation parameters from DWI-to-template registration were subsequently applied to co-register T1 and T2 images, ensuring spatial consistency across modalities. All MRI volumes were resampled to the template space using linear interpolation.

Lesion segmentation masks generated by the nnU-Net model were also transformed into the template space using the same transformation. Nearest-neighbor interpolation was applied for ROI masks to preserve discrete label values and avoid partial volume effects.

This standardized preprocessing pipeline enabled accurate voxel-wise alignment across modalities and facilitated reliable extraction of region-based imaging features for downstream analysis. All preprocessing procedures were conducted independently within each cross-validation training fold before model fitting.

### 2.5 Model development

Missing values were infrequent, and no variable had more than 5% missing values. Continuous variables with missing values were imputed using the median value calculated from the training data within each cross-validation fold, and categorical variables were imputed using the most frequent category. Imputation was performed before model fitting and without using information from the corresponding validation or test data.

#### 2.5.1 Image model

For each patient, three MRI modalities, including diffusion-weighted imaging (DWI), T1-weighted imaging (T1WI), and T2-fluid attenuated inversion recovery (T2-FLAIR), were used as inputs for the imaging-only model. The complete three-dimensional MRI volumes were directly used as model inputs without predefined region-of-interest selection or lesion-based cropping. The three modalities were arranged as a three-channel volumetric input tensor, with each channel corresponding to one MRI modality. Image intensities were normalized using patient-specific z-score normalization to reduce inter-subject intensity variability. No handcrafted imaging features were extracted, and the model directly learned high-level representations from the whole-brain MRI volumes.

A three-dimensional convolutional neural network (3D CNN)-based encoder was employed to automatically extract imaging representations from the volumetric MRI data. The encoder consisted of three sequential convolutional blocks. Each block contained a three-dimensional convolution layer, instance normalization, and rectified linear unit (ReLU) activation. Max pooling was applied after the first two convolutional blocks to reduce spatial dimensions. The numbers of convolutional filters were sequentially increased from 16 to 32 and finally to 64. An adaptive average pooling layer was subsequently used to generate a 64-dimensional imaging feature representation.

The extracted imaging features were subsequently passed through a fully connected classification head consisting of two linear layers (64 neurons and 1 neuron), ReLU activation, and dropout regularization (0.3). The final layer generated a single logit output, which was converted into an individualized SAP probability using sigmoid transformation during inference.

The imaging model was trained from scratch without pretrained weights. All parameters of the 3D CNN encoder and classification layers were optimized jointly. Model optimization was performed using the Adam optimizer with a learning rate of 1 × 10^−4^. Binary cross-entropy loss with logits (BCEWithLogitsLoss) was used as the loss function. The model was trained for 20 epochs with a batch size of 2.

A five-fold stratified cross-validation strategy was adopted to evaluate model performance while maintaining class distribution consistency. In each fold, the dataset was divided into training and validation sets using an 80:20 ratio. The model achieving the highest validation AUC during training was saved as the best-performing model for each fold. Out-of-fold predictions were generated by aggregating predictions from all validation subsets and were subsequently used for ROC analysis, calibration assessment, and decision curve analysis.

#### 2.5.2 Clinical model

A clinical model based on a multilayer perceptron (MLP) was developed using structured clinical variables alone to establish a non-imaging predictive baseline. Before model training, patient identifiers and imaging-related variables, including “ID,” “DWI,” “T1,” “T2,” and outcome labels, were excluded. The remaining clinical characteristics, comprising demographic information, vascular risk factors, and laboratory parameters, were used as model inputs. Brain-region features derived from the Automated Anatomical Labeling Atlas 3 (AAL3) were intentionally excluded from this model to evaluate the independent predictive contribution of conventional clinical information.

To reduce the impact of different feature scales and improve numerical stability during optimization, all clinical variables were standardized using z-score normalization. The normalization parameters were estimated only from the training data within each cross-validation iteration and subsequently applied to the corresponding validation/test data to prevent information leakage.

Model development and performance evaluation were performed using a nested five-fold stratified cross-validation framework. The outer five-fold stratified cross-validation was used for unbiased model assessment, in which four folds were used for model training and the remaining fold was reserved as an independent test set. Within each outer training cohort, an inner five-fold stratified cross-validation procedure was conducted to determine the optimal training epoch according to the highest validation AUC. After identification of the optimal epoch, the model was retrained using the complete outer training cohort and subsequently evaluated on the held-out outer test cohort. Predictions from all outer folds were aggregated to generate out-of-fold (OOF) probabilities, which were used for final model performance evaluation.

The clinical MLP consisted of two fully connected hidden layers containing 16 and 8 neurons, respectively. The first hidden layer was followed by batch normalization, a rectified linear unit (ReLU) activation function, and a dropout layer with a rate of 0.3. A second dropout layer was applied after the subsequent hidden layer to further reduce overfitting. The final output layer generated a single logit value for binary classification, and probabilities were obtained using the sigmoid activation function.

The model was optimized using the Adam optimizer with a learning rate of 0.001. Binary cross-entropy loss with logits was used as the optimization objective. L2 regularization was implemented through weight decay with a coefficient of 1 × 10^−4^. The model was trained with a batch size of 32, and the number of training epochs was adaptively determined through inner cross-validation, with a maximum search range of 50 epochs.

#### 2.5.3 Brain-region model

A brain-region-based machine learning model was developed using lesion location features derived from the Automated Anatomical Labeling Atlas 3 (AAL3). All brain-region features were obtained from the lesion localization results and represented the distribution of ischemic lesions across anatomical regions defined by the AAL3 atlas (Table 1).

**Table 1:** Top 10 brain regions with the highest stroke frequency.

Brain region	Frequency	Percentage(%)
Postcentral_L	97.00	22.77
Postcentral_R	78.00	18.31
Precentral_L	76.00	17.84
Precentral_R	65.00	15.26
Precuneus_R	65.00	15.26
Parietal_Inf_L	65.00	15.26
Cingulate_Mid_L	64.00	15.02
Parietal_Sup_L	64.00	15.02
Cingulate_Mid_R	61.00	14.32
Frontal_Sup_2_L	59.00	13.85

To obtain a robust assessment of model performance, a five-fold stratified cross-validation approach was adopted. The data were divided into five subsets while preserving class ratios. During each iteration, the model was trained on four subsets and validated on the remaining subset, with the process repeated five times so that every sample was used once for validation.

A logistic regression classifier was implemented using a pipeline consisting of feature standardization and classification. Continuous variables were normalized using z-score standardization via the StandardScaler. Logistic regression was selected because of its interpretability and robustness for structured high-dimensional data.

During each fold, the model was trained on the training subset and evaluated on the validation subset. Predicted probabilities for the positive class were recorded, and out-of-fold (OOF) predictions were aggregated across all folds to obtain unbiased estimates of model performance.

#### 2.5.4 Fusion model

A multimodal deep learning framework was developed to integrate volumetric MRI representations with structured clinical and anatomical characteristics for individualized prediction of stroke-associated pneumonia (SAP). The framework consisted of two parallel branches, including an imaging representation branch and a clinical–anatomical feature processing branch, followed by feature-level integration for final classification.

The imaging branch was designed to process three-dimensional multimodal MRI volumes, including DWI, T1WI, and T2-FLAIR. For each patient, the three MRI modalities were stacked as a three-channel volumetric input tensor, with each channel representing one MRI modality and preserving its corresponding anatomical and pathological information. Image intensities were normalized using patient-specific z-score normalization to reduce inter-subject intensity variability and improve training stability. No predefined region-of-interest selection or handcrafted imaging feature extraction was performed, allowing the network to directly learn high-level imaging representations from the entire MRI volumes.

A three-dimensional convolutional neural network (3D CNN)-based encoder was developed to automatically extract hierarchical imaging features from the three MRI modalities (DWI, T1WI, and T2-FLAIR), which were organized as a three-channel 3D input. The encoder consisted of three sequential convolutional blocks, each comprising a 3D convolutional layer with a 3 × 3 × 3 kernel, stride of 1, and padding of 1, followed by instance normalization and rectified linear unit (ReLU) activation. The numbers of convolutional filters in the three blocks were 16, 32, and 64, respectively. A 2 × 2 × 2 max-pooling layer with a stride of 2 was applied after the first two blocks to progressively reduce the spatial dimensions. Finally, adaptive average pooling was applied to obtain a 64-dimensional latent imaging representation.

The clinical–anatomical feature branch incorporated structured patient-level information, including demographic characteristics, vascular risk factors, laboratory measurements, and lesion-related brain-region features derived from the Automated Anatomical Labeling Atlas 3 (AAL3). These variables were processed through a fully connected layer to generate a 32-dimensional clinical–anatomical representation. The extracted imaging representation and clinical–anatomical representation were subsequently concatenated to achieve feature-level multimodal fusion, enabling complementary information from MRI-derived deep features and structured clinical–anatomical characteristics to contribute jointly to SAP prediction.

The fused representations were passed through a fully connected classification module consisting of two linear layers (128 and 64 neurons), ReLU activation, and dropout regularization (0.3). The final linear layer generated a single logit output, which was transformed into an individualized SAP probability using sigmoid transformation during inference.

The model was trained from scratch without external pretrained weights. All parameters of the imaging encoder and classification layers were optimized jointly during training. Optimization was performed using the Adam optimizer with an initial learning rate of 1 × 10^−4^. Binary cross-entropy loss with logits (BCEWithLogitsLoss) was used as the objective function. The batch size was set to 2, and training was performed for a maximum of 20 epochs.

To obtain unbiased performance estimates and reduce potential overfitting, a nested five-fold stratified cross-validation strategy was adopted. In the outer loop, the dataset was divided into five stratified folds, with four folds used for model development and the remaining fold reserved as an independent test set. Within each outer training cohort, an inner five-fold stratified cross-validation procedure was conducted to determine the optimal number of training epochs according to the highest validation AUC. After epoch selection, the model was retrained using the complete outer training cohort and evaluated on the corresponding held-out outer test cohort.

Random seeds were fixed before all experiments to ensure reproducibility of data partitioning, model initialization, and optimization procedures. Out-of-fold predictions from the outer test cohorts were aggregated for subsequent performance evaluation, including ROC analysis, calibration assessment, and decision curve analysis.

## 3 Patient characteristics

A total of 426 patients were included in the final analysis, among whom 71 patients (16.67%) developed stroke-associated pneumonia (SAP) (Table 2).

**Table 2:** The clinical characteristics of all patients.

Clinical feature	Lung infections(−) (*n* = 355)	Lung infections(+) (*n* = 71)	*p*-value
Age	65.75 ± 11.02	73.61 ± 10.29	<0.001
Neutrophils%	65.12 ± 9.59	75.18 ± 10.13	<0.001
Lymphocyte%	24.84 ± 8.03	16.19 ± 8.11	<0.001
Monocyte%	7.60 ± 2.24	6.97 ± 2.31	0.33
Eosinophils%	2.03 ± 1.81	1.33 ± 1.57	0.003
Basophils%	0.42 ± 0.26	0.33 ± 0.21	0.009
Neutrophils	4.41 (3.54–5.61)	5.89 (4.34–9.04)	<0.001
Lymphocyte	1.71 ± 0.64	1.29 ± 0.56	<0.001
Monocyte	0.53 ± 0.18	0.58 ± 0.24	0.035
Eosinophils	0.14 ± 0.12	0.10 ± 0.11	0.009
Basophils	0.03 ± 0.02	0.03 ± 0.02	0.486
Fibrinogen	3.03 (2.57–3.48)	3.35 (2.76–4.35)	0.02
D_dimer	0.37 (0.23–0.74)	0.96 (0.43–2.62)	0.07
FDP	1.27 (0.4–2.45)	3.10 (1.10–8.30)	0.001
Sex			0.196
Male	243 (68.5)	43 (60.6)	
Female	112 (31.5)	28 (39.4)	
Hypertension			0.027
Yes	205 (57.7)	51 (71.8)	
No	150 (42.3)	20 (28.2)	
Cardiopathy			0.062
Yes	45 (12.7)	15 (21.1)	
No	310 (87.3)	56 (78.9)	
Ischemic stroke			0.095
Yes	56 (15.8)	17 (23.9)	
No	299 (84.2)	54 (76.1)	
Diabetes			0.621
Yes	90 (25.4)	20 (28.2)	
No	265 (74.6)	51 (71.8)	
Smoke			0.014
Yes	117 (33.0)	13 (18.3)	
No	238 (67.0)	58 (81.7)	

Values are given as or mean± SD. FDP, Fibrinogen degradation products.

The clinical characteristics of patients with and without SAP are summarized in Table 2. Patients in the SAP group were significantly older than those in the non-SAP group (73.61 ± 10.29 vs. 65.75 ± 11.02 years, *p* < 0.001).

Regarding inflammatory markers, the SAP group exhibited significantly higher neutrophil percentage (75.18 ± 10.13% vs. 65.12 ± 9.59%, *p* < 0.001) and lower lymphocyte percentage (16.19 ± 8.11% vs. 24.84 ± 8.03%, *p* < 0.001). Similarly, absolute neutrophil counts were elevated [5.89 (4.34–9.04) vs. 4.41 (3.54–5.61), *p* < 0.001], while lymphocyte counts were significantly reduced (1.29 ± 0.56 vs. 1.71 ± 0.64, *p* < 0.001) in the SAP group.

In terms of hematological parameters, both eosinophil percentage and eosinophil counts were significantly lower in the SAP group (*p* = 0.003 and *p* = 0.009, respectively), whereas monocyte counts were slightly elevated (*p* = 0.035). No significant difference was observed in basophil counts between the two groups (*p* = 0.486).

Regarding coagulation-related indicators, fibrinogen and fibrin degradation products (FDP) were significantly higher in patients with SAP (*p* = 0.02 and *p* = 0.001, respectively), while D-dimer showed an increasing trend without statistical significance (*p* = 0.07).

Among clinical risk factors, hypertension was more frequently observed in the SAP group (71.8% vs. 57.7%, *p* = 0.027), smoking prevalence was lower in the SAP group (18.3% vs. 33.0%, *p* = 0.014). No significant differences were found in sex, cardiopathy, prior ischemic stroke history, or diabetes mellitus (all *p* > 0.05).

## 4 Results

### 4.1 Model performance, clinical utility, and validation

The performance of the clinical, brain region, deep learning (DL), and fusion models is shown in Figure 3. The fusion model achieved the highest discriminative performance in this internally validated cohort, with an AUC of 0.782 (95% CI: 0.712–0.839), followed by the clinical model (AUC = 0.756), the DL model (AUC = 0.693), and the brain region model (AUC = 0.501) (Table 3).

**Figure 3:**
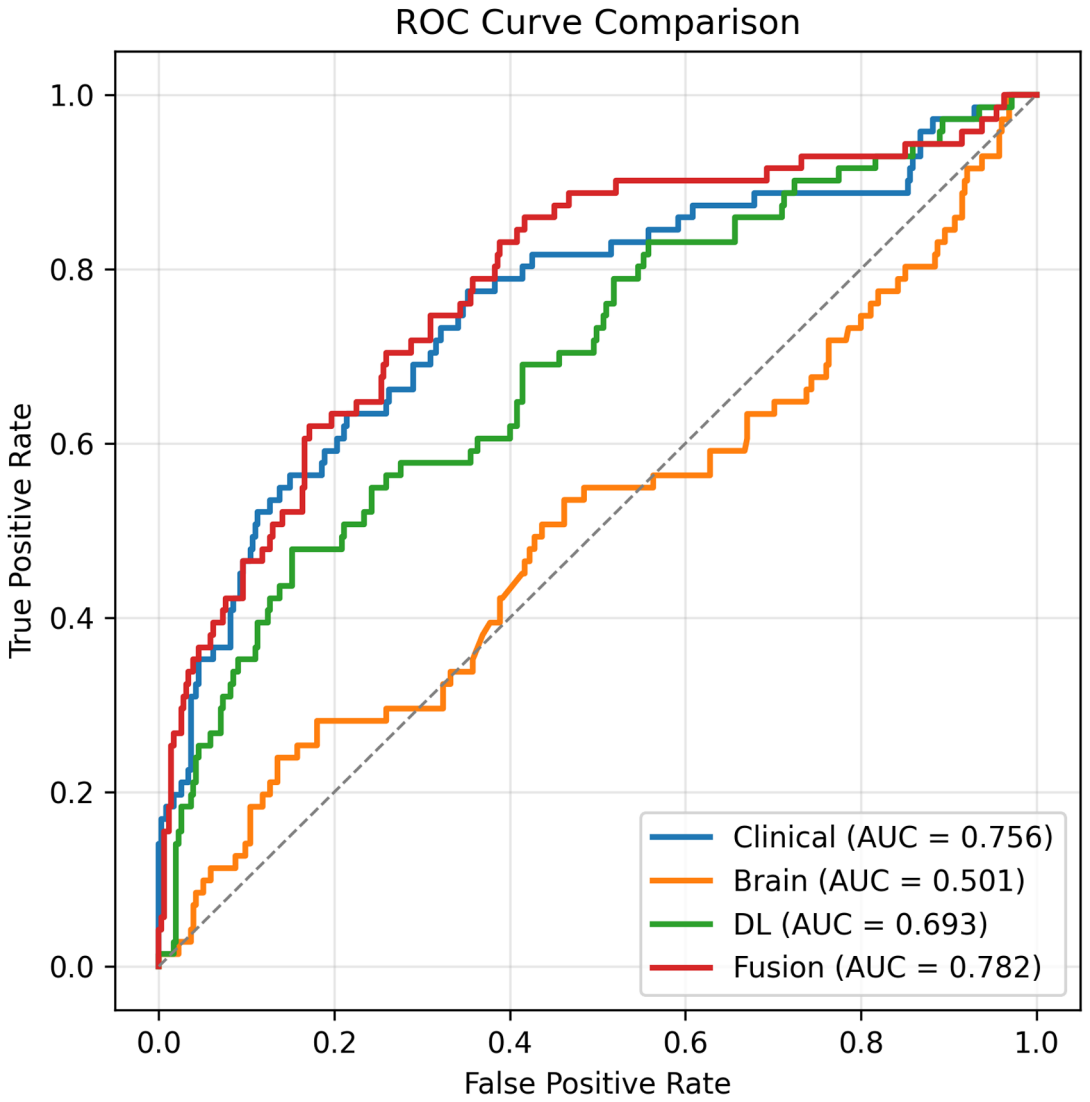
Multimodal feature extraction and fusion architecture. Imaging features are learned using a 3D CNN, clinical features are processed via fully connected layers, and brain region involvement (AAL3 atlas, 170 regions) is encoded as a binary vector and modeled using logistic regression. Features from all modalities are fused at the feature level to generate the final prediction.

**Table 3:** Predictive performance of different models for SAP.

Model	AUC (95% CI)	Accuracy	Sensitivity	Specificity	Precision	F1-score	PPV	NPV	Threshold
Clinical	0.756 (0.682–0.820)	0.67	0.78	0.65	0.31	0.44	0.31	0.94	0.16
Brain	0.501 (0.423–0.579)	0.76	0.24	0.87	0.26	0.25	0.26	0.85	0.22
DL	0.693 (0.623–0.763)	0.79	0.48	0.85	0.39	0.43	0.39	0.89	0.20
Fusion	0.782 (0.712–0.839)	0.79	0.62	0.83	0.42	0.50	0.42	0.92	0.17

Brain-region features alone showed limited predictive capability but were retained as an independent modality to evaluate whether anatomical localization provided incremental information when integrated into multimodal learning. The ROC curves demonstrated that the fusion model consistently achieved superior sensitivity-specificity trade-offs across nearly all threshold levels.

The fusion model showed incremental improvement over individual modalities, suggesting the potential benefit of multimodal integration. The fusion model improved AUC by approximately 0.026 compared with the clinical model, 0.089 compared with the DL model, and 0.281 compared with the brain region model, indicating that multimodal integration provided additional predictive information beyond individual models.

These differences were statistically validated using the DeLong test. The fusion model showed a numerical improvement over the clinical model, although the difference did not reach statistical significance (*p* = 0.298). Significant differences were observed between the fusion model and the DL model (*p* < 0.001) and brain-region model (*p* < 0.001). Although the clinical model showed higher AUC than the DL model, the difference was not statistically significant (*p* = 0.121). The brain region model exhibited near-random discrimination, indicating that region-level features alone are insufficient for accurate prediction in this task.

From a clinical decision-making standpoint, decision curve analysis (DCA) showed that the fusion model achieved the greatest net benefit over a wide range of threshold probabilities (approximately 0.05–0.50), suggesting strong clinical utility (Figure 4). The clinical model also showed consistent benefit, particularly at lower thresholds, supporting its role as a stable baseline model. The DL model provided limited but non-negligible benefit within a narrower threshold range, whereas the brain region model showed minimal or negative net benefit across most thresholds, further confirming its limited clinical utility. The 95% confidence intervals (CIs) of the net benefit curves were estimated using 1,000 bootstrap resamples and are presented as shaded areas around the corresponding decision curves. These indicate that the fusion model provides greater clinical value in identifying patients at high risk of post-stroke pulmonary infection.

**Figure 4:**
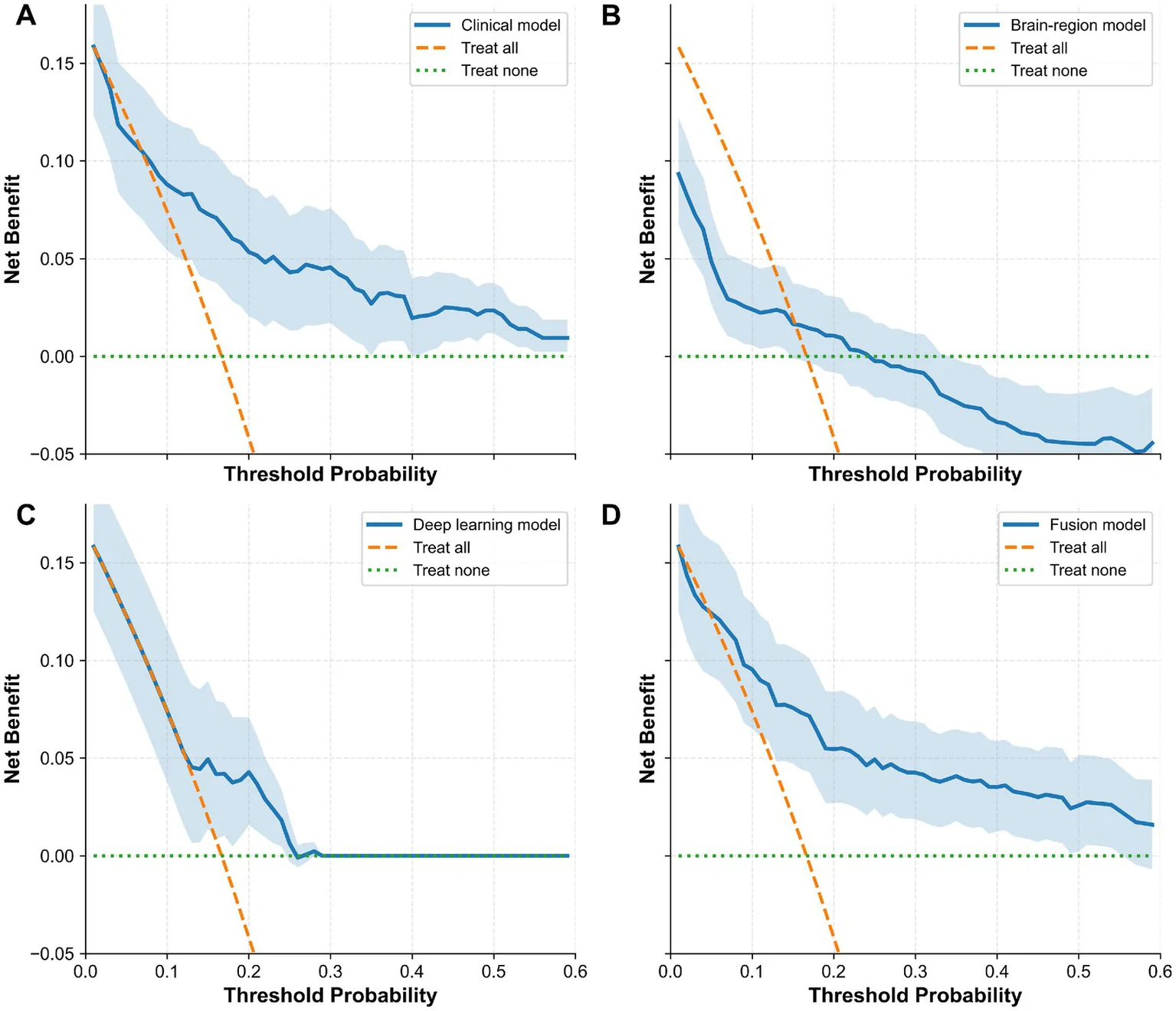
Decision curve analysis of different models. **(A–D)** DCA curves of the clinical, brain region, DL, and fusion models. Shaded areas indicate the 95% confidence intervals. “Treat-all” and “treat-none” are shown as reference lines.

The clinical model also showed relatively stable net benefit, particularly at lower threshold probabilities, suggesting its usefulness as a baseline predictive tool in early screening scenarios. In contrast, the deep learning model exhibited moderate net benefit within a narrower threshold range, indicating limited standalone clinical applicability despite its ability to capture imaging features.

Notably, the brain region model demonstrated minimal or even negative net benefit across most threshold probabilities, frequently overlapping with or falling below the “treat-all” strategy. This finding suggests that relying solely on coarse atlas-based regional features is insufficient for clinical decision-making.

Importantly, the fusion model consistently showed superior performance compared with both the “treat-all” and “treat-none” strategies across clinically relevant threshold probabilities. At intermediate risk thresholds—where clinical decisions are most critical—the advantage of the fusion model was particularly pronounced, indicating its potential to reduce unnecessary interventions while improving early detection of high-risk patients.

Calibration performance was further evaluated using quantile-based calibration curves with five probability groups, together with Brier scores, calibration intercepts, calibration slopes, and bootstrap-derived 95% confidence intervals (Figure 5). The fusion model demonstrated favorable calibration performance, with a Brier score of 0.111. The calibration intercept was 0.111 (95% CI: −0.349 to 0.555), indicating limited overall prediction bias, while the calibration slope was 0.900 (95% CI: 0.682–1.162), suggesting good agreement between predicted and observed risks. Although the Hosmer–Lemeshow test indicated a statistically significant deviation from perfect calibration (*p* = 0.011), the calibration intercept and slope suggested that the magnitude of miscalibration was relatively limited. The clinical model also showed acceptable calibration, although deviations from the ideal calibration line became more apparent at higher predicted probabilities, indicating reduced reliability in estimating patients with higher SAP risk. The DL model exhibited moderate calibration performance, with greater fluctuations and potential overestimation in intermediate probability ranges. Overall, the fusion model provided more reliable probability estimation and improved risk stratification capability compared with individual models, supporting its potential value for individualized SAP prediction.

**Figure 5:**
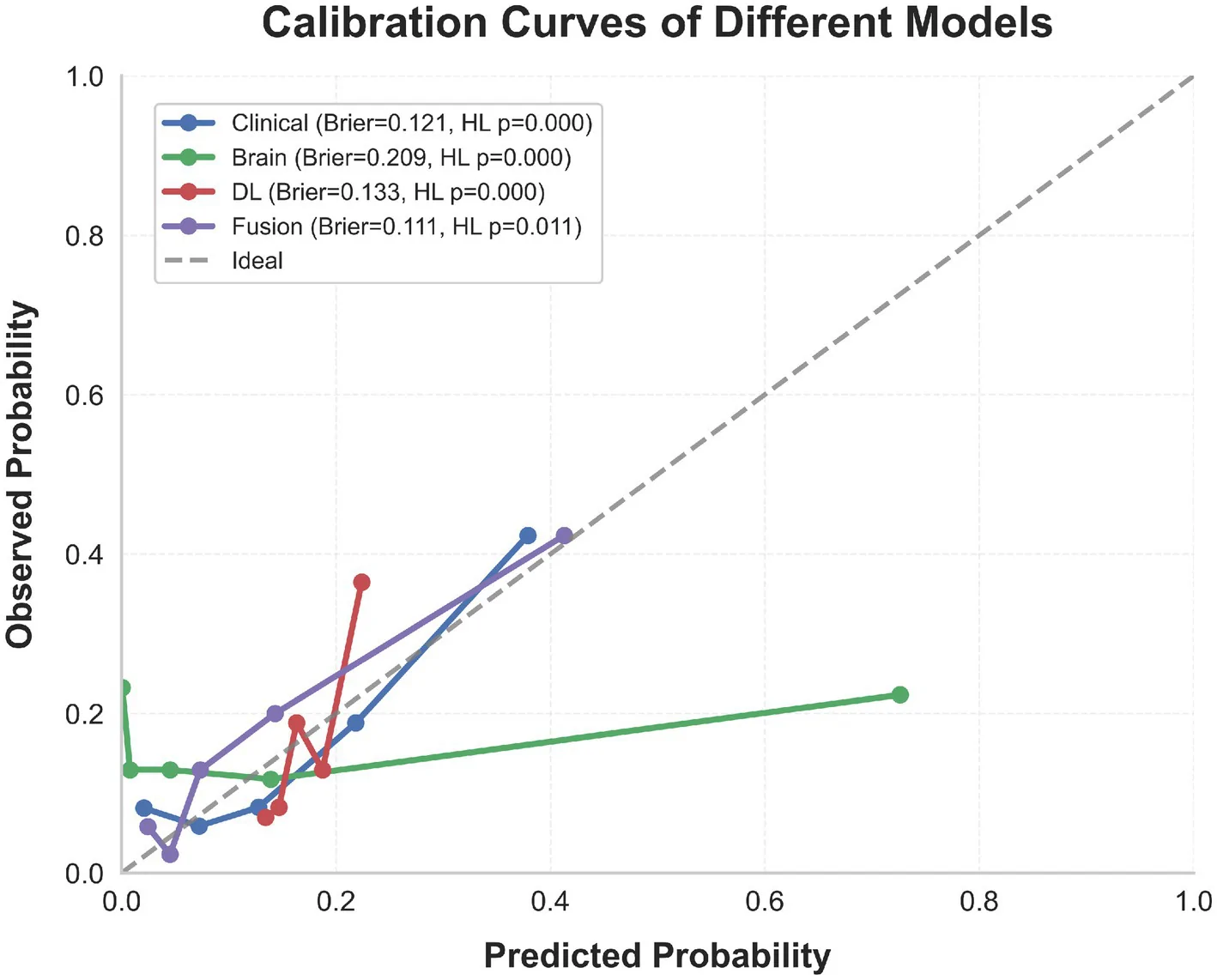
Calibration curves of different models for predicting SAP. The curves demonstrate the agreement between predicted probabilities and observed outcomes, with the diagonal line representing perfect calibration. Calibration performance was evaluated using calibration curves, Brier scores, and the Hosmer–Lemeshow goodness-of-fit test.

### 4.2 Model interpretability

To enhance interpretability, Grad-CAM was applied to visualize the spatial regions contributing to model predictions (Figure 6). The activation maps demonstrated that the deep learning model focused on lesion-related areas across multimodal MRI inputs (DWI, T1, and T2), with high-intensity responses localized to infarct regions and their surrounding cortical and subcortical structures.

**Figure 6:**
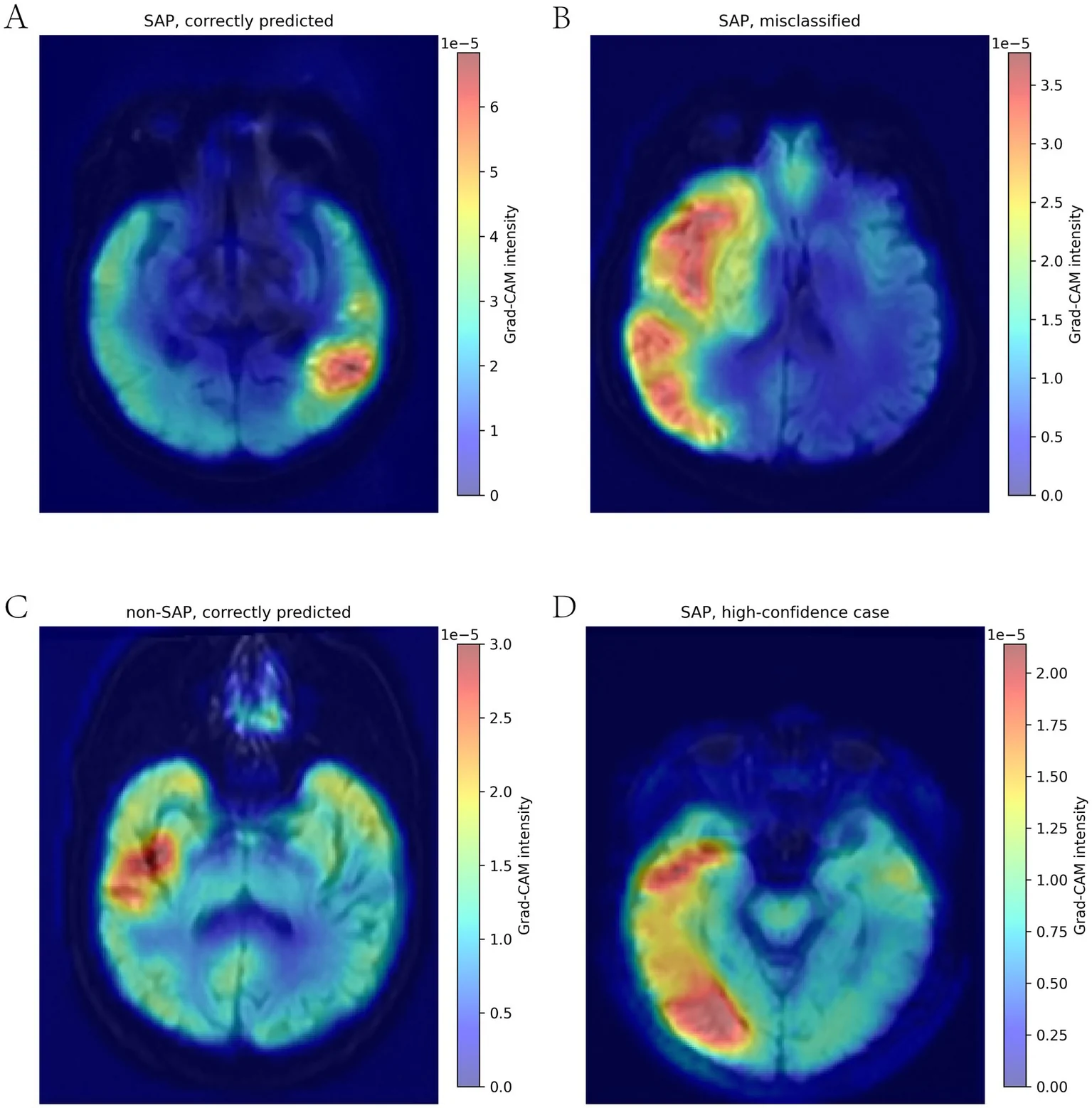
Representative Grad-CAM visualization of the multimodal deep learning model for predicting SAP. Representative Grad-CAM heatmaps illustrate the regions contributing to model predictions in different prediction scenarios. **(A)** An SAP case correctly predicted by the model, showing prominent activation in the ischemic lesion region. **(B)** An SAP case that was misclassified, with relatively extensive and heterogeneous activation predominantly involving the ischemic hemisphere. **(C)** A non-SAP case correctly classified by the model, with activation mainly localized to the lesion-related region. **(D)** A high-confidence SAP case, demonstrating strong and spatially concentrated Grad-CAM activation in the affected hemisphere. Warmer colors indicate higher Grad-CAM intensity and therefore greater contribution to the model prediction.

Notably, these regions overlapped with anatomically and functionally relevant brain areas, including cortical regions associated with motor and autonomic regulation. The model attention was concentrated on biologically plausible regions rather than diffuse or irrelevant background signals, indicating that the model captured meaningful pathological features rather than spurious correlations.

In several cases, additional activation was observed in regions beyond the primary lesion, suggesting that the model may capture network-level alterations or secondary effects following stroke. This pattern supports the hypothesis that post-stroke pulmonary infection may be associated with distributed brain network dysfunction rather than focal injury alone.

## 5 Discussion

In this study, we developed and validated a multimodal fusion model integrating clinical variables, brain region features, and deep learning–derived imaging representations to predict post-stroke pulmonary infection. Previous computational models for SAP prediction mainly relied on clinical variables or single-modality imaging, whereas our framework integrates multimodal MRI representations, anatomical information, and clinical characteristics (Table 4).

**Table 4:** Comparison of previous and proposed SAP models.

Feature	Qiao et al. (49)	Xie et al. (47)	This study
Clinical variables	✓	✓	✓
Medical imaging	CT	✗	Multimodal MRI (DWI/T1/T2)
Deep learning	✓	✗	✓ (3D CNN)
Brain-region encoding	✗	✗	✓ (AAL3)
Multimodal fusion	CT + Clinical	✗	MRI + Clinical + Brain region
Explainable AI	✗	SHAP	Grad-CAM
Target population	ICH	AIS	AIS

The superior performance of the fusion model highlights the value of multimodal integration (22, 30). While the clinical model suggested relatively strong predictive ability, it primarily reflects systemic physiological and inflammatory status (23). In contrast, imaging-based models capture structural and spatial information related to brain injury (13, 14). Although the overall performance of the brain-region model was relatively limited, significant differences were still observed in the lesion-involved brain regions between patients with stroke-associated pneumonia and those without pneumonia (Table 5). Meanwhile, the fusion model suggested that anatomical information may provide complementary context when combined with imaging and clinical features. The observed improvement of the fusion model suggests that post-stroke pulmonary infection is not determined by a single domain, but rather results from the interaction between systemic factors and brain injury patterns. This finding is consistent with the concept of the brain–lung axis, in which central nervous system injury can induce systemic immunosuppression and increase susceptibility to infection (7, 33). Previous studies have shown that stroke can trigger stroke-induced immunodepression syndrome (SIDS), characterized by lymphocyte dysfunction and altered cytokine responses, ultimately predisposing patients to pulmonary infections (7, 16). Furthermore, because a relatively large number of atlas-based regions were evaluated with a limited number of SAP events, the possibility of overfitting and multiple-comparison bias cannot be completely excluded. Therefore, the identified regional associations should be interpreted as exploratory findings requiring validation in larger cohorts.

**Table 5:** Top 10 brain regions with the highest stroke frequency of SAP patient.

Brain region	Lung infections (+) n (%)	Lung infections (−) n (%)	*p*-value
Precentral_L	19 (26.76%)	41 (11.55%)	<0.001
Insula_L	19 (26.76%)	38 (10.70%)	<0.001
Postcentral_L	19 (26.76%)	36 (10.14%)	<0.001
Precuneus_L	17 (23.94%)	29 (8.17%)	<0.001
Precuneus_R	17 (23.94%)	25 (7.04%)	<0.001
Parietal_Inf_L	16 (22.54%)	31 (8.73%)	<0.001
Frontal_Mid_2_L	15 (21.13%)	27 (7.61%)	<0.001
Rolandic_Oper_L	15 (21.13%)	24 (6.76%)	<0.001
Caudate_L	14 (19.71%)	22 (6.20%)	<0.001
Frontal_Sup_2_L	14 (19.71%)	26 (7.32%)	<0.001
Temporal_Mid_L	14 (19.71%)	21 (5.92%)	<0.001
Putamen_L	14 (19.71%)	23 (6.48%)	<0.001

Another limitation is that excluding patients with severe MRI motion artifacts or inadequate image quality may have introduced selection bias. Although this exclusion was necessary to ensure reliable imaging analysis, it may have resulted in a cohort with better-quality MRI data than those encountered in routine practice, potentially limiting the generalizability of the model to patients with suboptimal imaging quality. Future multicenter studies should evaluate model robustness across more heterogeneous imaging conditions.

Interestingly, the clinical model outperformed the deep learning model when used alone, supporting the possibility that traditional clinical variables remain highly informative. Variables such as age, inflammatory markers, and coagulation indicators likely reflect systemic susceptibility to infection (23). However, the deep learning model provided additional spatial context by identifying lesion-related features that are not explicitly captured by structured data (14). The improvement observed after fusion suggests that these two types of information are complementary rather than redundant.

Compared with conventional SAP scoring systems such as A^2^DS^2^ and ISAN, which are based exclusively on clinical variables, our multimodal framework additionally incorporates MRI-derived imaging features and lesion-aware neuroanatomical information, enabling a more comprehensive characterization of SAP risk. Although a direct comparison with these scoring systems was beyond the scope of the present study, our findings suggest that multimodal integration may offer additional predictive value for individualized risk assessment.

Notably, many of these regions are closely associated with motor control, somatosensory processing, and autonomic regulation. The precentral and postcentral gyri are involved in voluntary motor function and sensorimotor integration, which are essential for coordinated swallowing and airway protection. Damage to these regions may contribute to dysphagia and increase the risk of aspiration pneumonia (15, 34, 35).

The insular cortex, one of the most frequently involved regions in our cohort, plays a critical role in autonomic nervous system regulation and immune modulation. Previous studies have suggested that insular damage is associated with altered sympathetic activity and increased susceptibility to infection (7). In addition, regions such as the precuneus and inferior parietal lobule are key components of large-scale brain networks and are involved in higher-order integration and network connectivity. Lesions affecting these regions may reflect more extensive brain injury and contribute to systemic dysfunction through network-level disruption.

Furthermore, the involvement of subcortical structures, including the caudate nucleus and putamen, suggests that disruption of cortico-subcortical circuits may also play a role in the development of SAP (36). These findings collectively support the hypothesis that post-stroke pulmonary infection is associated with both focal damage in functionally critical regions and distributed network-level alterations, rather than being driven by isolated lesions alone (37).

In contrast, the brain region model based on atlas-level features showed poor predictive performance. This may be attributed to the limited granularity of region-based encoding, which reduces complex lesion patterns into simplified regional involvement (38). Such an approach may fail to capture subtle spatial heterogeneity and lesion morphology, both of which are critical for understanding post-stroke complications (13). These findings suggest that coarse anatomical representations alone are insufficient for accurate prediction and should be combined with more detailed imaging features.

From a clinical perspective, decision curve analysis suggested that the fusion model provided the greatest net benefit across a wide range of threshold probabilities, supporting its potential utility in real-world decision-making (39). In practical settings, such as early-stage hospitalization or intensive care unit (ICU) monitoring, this model could assist clinicians in identifying high-risk patients at an early stage, thereby facilitating timely preventive strategies such as intensified respiratory management, infection surveillance, and individualized treatment planning (40–42). Notably, the model maintained positive net benefit compared with both “treat-all” and “treat-none” strategies, suggesting that it may help reduce unnecessary interventions while improving early detection of pulmonary infection (39).

In addition, the good calibration performance of the fusion model indicates that its predicted probabilities are reliable, which is essential for risk stratification and clinical adoption (39). Accurate probability estimation allows clinicians not only to classify patients into risk categories but also to quantify individualized risk, thereby supporting precision medicine approaches.

The Grad-CAM analysis further enhanced the interpretability of the deep learning model by revealing that the network primarily focused on lesion-related regions (28). These regions were frequently located in cortical and subcortical areas, including regions associated with motor and autonomic function. Previous studies have suggested that damage to specific brain regions, such as the insula and motor cortex, may disrupt autonomic regulation and immune responses, thereby increasing susceptibility to infection (3, 7, 43, 44). The spatial patterns observed in our study are consistent with these findings and provide additional support for the involvement of distributed brain networks in post-stroke pulmonary infection.

Interestingly, in some cases, the model also highlighted regions beyond the primary lesion, suggesting that secondary network-level changes may contribute to infection risk. This observation aligns with emerging evidence that stroke induces widespread functional and immunological alterations, rather than being limited to focal damage. Therefore, the ability of deep learning models to capture such complex spatial patterns may represent an important advantage over traditional feature-based approaches (14).

Several limitations of this study should be acknowledged. First, this was a single-center retrospective study, which may limit the generalizability of the findings. Because this study was conducted at a single center using retrospective data and internally validated through five-fold cross-validation, the generalizability of the proposed model remains uncertain. Future multicenter prospective studies with independent external validation cohorts are required before routine clinical implementation. Therefore, the present results should be considered exploratory evidence based on internal validation rather than definitive proof of clinical effectiveness. Second, although the deep learning model suggested interpretability through Grad-CAM, the underlying biological mechanisms cannot be fully established based on imaging data alone. Future studies integrating immunological or molecular biomarkers (e.g., cytokines, immune cell profiling) may provide deeper insights into the brain–lung interaction (7, 18, 45–48). Finally, prospective studies are warranted to evaluate whether model-guided interventions can improve clinical outcomes, such as reducing infection incidence, shortening hospital stay, or improving functional recovery.

It is declared that none of the authors have any conflicts of interest.

## Data Availability

The raw data supporting the conclusions of this article will be made available by the authors, without undue reservation.
